# Effects of Exercise Training on Circulating and Skeletal Muscle Renin-Angiotensin System in Chronic Heart Failure Rats

**DOI:** 10.1371/journal.pone.0098012

**Published:** 2014-05-23

**Authors:** Igor Lucas Gomes-Santos, Tiago Fernandes, Gisele Kruger Couto, Julio César Ayres Ferreira-Filho, Vera Maria Cury Salemi, Fernanda Barrinha Fernandes, Dulce Elena Casarini, Patricia Chakur Brum, Luciana Venturini Rossoni, Edilamar Menezes de Oliveira, Carlos Eduardo Negrao

**Affiliations:** 1 Heart Institute (InCor-HCFMUSP), University of São Paulo Medical School, São Paulo, Brazil; 2 School of Physical Education and Sport, University of São Paulo, São Paulo, Brazil; 3 Department of Physiology and Biophysics, Institute of Biomedical Science, University of São Paulo, São Paulo, Brazil; 4 Division of Nephrology, Kidney and Hypertension Hospital, Federal University of São Paulo, São Paulo, Brazil; Lerner Research Institute, United States of America

## Abstract

**Background:**

Accumulated evidence shows that the ACE-AngII-AT1 axis of the renin-angiotensin system (RAS) is markedly activated in chronic heart failure (CHF). Recent studies provide information that Angiotensin (Ang)-(1–7), a metabolite of AngII, counteracts the effects of AngII. However, this balance between AngII and Ang-(1–7) is still little understood in CHF. We investigated the effects of exercise training on circulating and skeletal muscle RAS in the ischemic model of CHF.

**Methods/Main Results:**

Male Wistar rats underwent left coronary artery ligation or a Sham operation. They were divided into four groups: 1) Sedentary Sham (Sham-S), 2) exercise-trained Sham (Sham-Ex), sedentary CHF (CHF-S), and exercise-trained CHF (CHF-Ex). Angiotensin concentrations and ACE and ACE2 activity in the circulation and skeletal muscle (soleus and plantaris) were quantified. Skeletal muscle ACE and ACE2 protein expression, and AT1, AT2, and Mas receptor gene expression were also evaluated. CHF reduced ACE2 serum activity. Exercise training restored ACE2 and reduced ACE activity in CHF. Exercise training reduced plasma AngII concentration in both Sham and CHF rats and increased the Ang-(1–7)/AngII ratio in CHF rats. CHF and exercise training did not change skeletal muscle ACE and ACE2 activity and protein expression. CHF increased AngII levels in both soleus and plantaris muscle, and exercise training normalized them. Exercise training increased Ang-(1–7) in the plantaris muscle of CHF rats. The AT1 receptor was only increased in the soleus muscle of CHF rats, and exercise training normalized it. Exercise training increased the expression of the Mas receptor in the soleus muscle of both exercise-trained groups, and normalized it in plantaris muscle.

**Conclusions:**

Exercise training causes a shift in RAS towards the Ang-(1–7)-Mas axis in skeletal muscle, which can be influenced by skeletal muscle metabolic characteristics. The changes in RAS circulation do not necessarily reflect the changes occurring in the RAS of skeletal muscle.

## Introduction

Increased renin angiotensin system (RAS) has been consistently documented in chronic heart failure (CHF). Plasma Angiotensin (Ang) II levels are increased in CHF [1]–[4]. Likewise, RAS elevation in the heart, kidneys, and brain has been reported in CHF [5]–[8]. This scenario shows that CHF is characterized by overall RAS activation.

The classic RAS cascade depends on ACE-mediated conversion of AngI into AngII, an octapeptide binding to AT1 receptor. This ACE-AngII-AT1 axis contributes to cardiac remodeling [5], [9], high sympathetic activity [7], [10], and impaired vasodilation [11] in CHF. In addition, RAS exacerbation plays a role in skeletal myopathy, including reactive oxygen species generation, protein degradation, and apoptosis [12]–[14]. Recent studies provide evidence of an endogenous RAS axis that counterbalances the ACE-AngII-AT1 effects. ACE2, an ACE homolog enzyme [15], [16], metabolizes AngII into the heptapeptide Ang-(1–7) [17], which in turn binds to the Mas receptor [18]. This ACE2-Ang-(1–7)-Mas axis causes vasodilation and improves skeletal muscle metabolism [19], [20]. In addition, ACE2-Ang-(1–7)-Mas axis has antifibrotic and antiapoptotic properties, opposing the deleterious effects of ACE-AngII-AT1 overactivity [21].

Previous studies have shown that exercise training is a remarkable strategy in the treatment of CHF. This therapy reduces sympathetic nerve activity and vasoconstriction [22], [23]. In addition, exercise training significantly increases exercise tolerance and quality of life in CHF patients [24]. Effects of exercise training on RAS have also been reported. Exercise training reduces plasma AngII levels [10], [25]. In the cardiac muscle, exercise reduces ACE concentration, which may contribute to the reduction in hypertrophy and collagen deposition in rats with myocardial infarction [5]. More recently, we found a decrease in ACE and AngII concentrations and an increase in ACE2 and Ang-(1–7) concentration in cardiac muscle in exercise-trained rats [26]. In addition, exercise training has been shown to increase cardiac Ang-(1–7) in spontaneous hypertensive rats [27] and to normalize brain ACE and ACE2 expression in CHF rabbits [7]. However, the effects of exercise training on RAS in skeletal muscle are unknown.

To improve the understanding regarding the role of exercise training on circulating and skeletal muscle RAS, we investigated the effects of exercise training on ACE-AngII-AT1 and ACE2-Ang-(1–7)-Mas axis in rats with CHF. Our hypotheses were that: 1) Exercise training would shift the balance of RAS towards the ACE2-Ang-(1–7)-Mas axis; 2) The changes in skeletal muscle RAS would be independent of those observed in the circulation; and 3) The effects of exercise training on skeletal muscle would be influenced by muscle metabolic characteristic.

## Methods

### Animals

**Figure 1:**
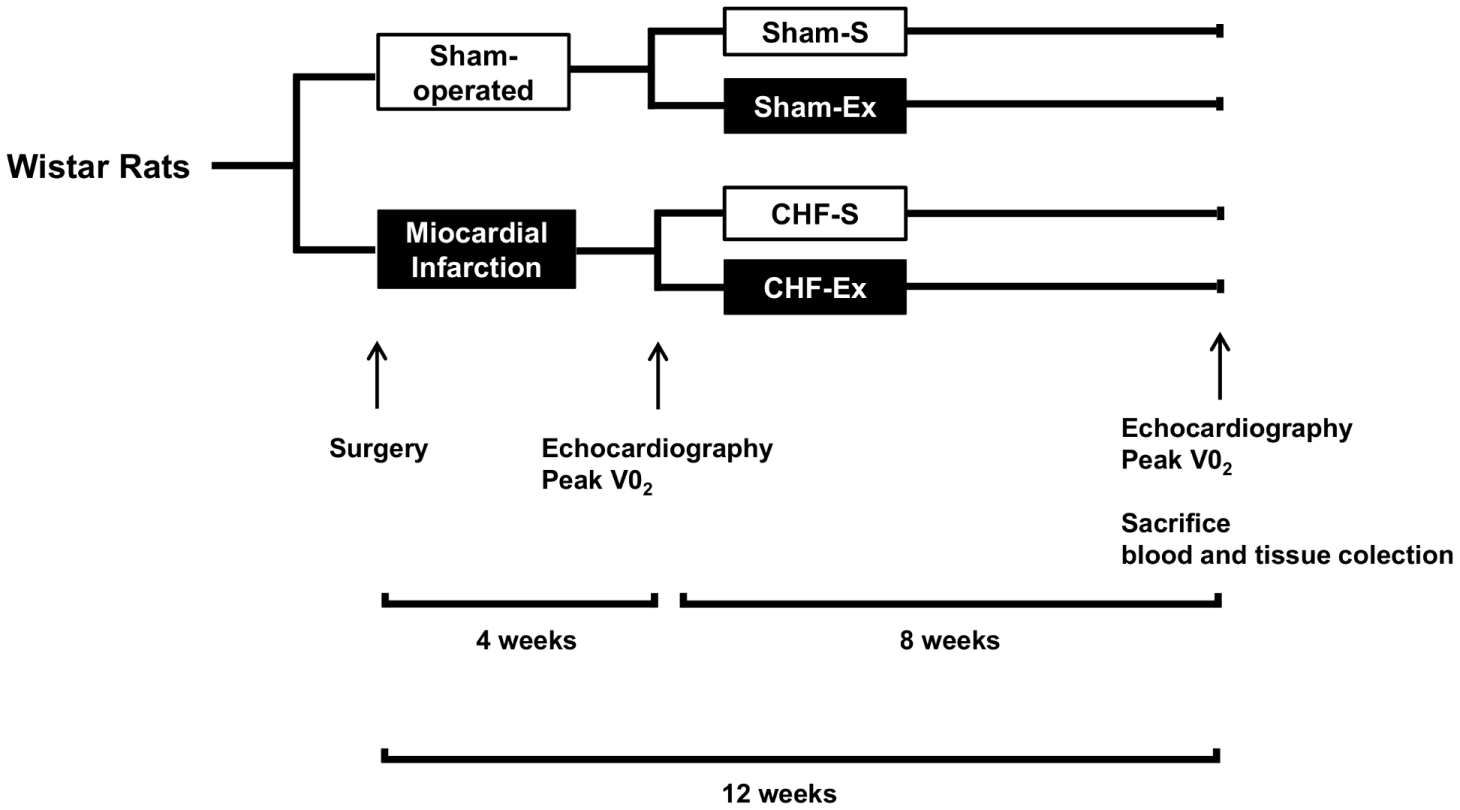
Experimental protocol.

Two-month-old, male, Wistar rats from the Multidisciplinary Center for Biological Investigation on Laboratory Animal Science (CEMIB), University of Campinas, underwent surgical occlusion of the main descending branch of the left coronary artery or fictitious myocardial infarction (Sham). After a 4-week period, they were subdivided into four groups: 1) Sedentary sham-operated (Sham-S, n = 10); 2) exercise-trained sham-operated (Sham-Ex, n = 10), 3) sedentary chronic heart failure (CHF-S, n = 12); and 4) exercise-trained chronic heart failure (CHF-Ex, n = 12). Then, basal echocardiography was performed and peak oxygen consumption was measured. Finally, exercise training or follow-up began (Experimental protocol, Fig. 1). The rats were kept 3–4 per cage in a temperature-controlled room with a 12∶12-hour light-dark cycle. They were fed with a standard laboratory diet and water *ad libitum*. The study was approved by the Scientific Committee of the Heart Institute (InCor-HCFMUSP) and Ethical Committee for Research Protocols of the University of São Paulo Medical School (CAPPesq 149/09). The study was carried out in accordance with the recommendations in the Guide for the Care and Use of Laboratory Animals of the National Institutes of Health, USA. The surgery and echocardiography was performed under anesthesia, and all efforts were made to minimize suffering.

### Myocardial Infarction Induction

The rats were anesthetized with a single injection of ketamine (50 mg.kg^−1^ body weight, Parke-Davis) and xylazine (10 mg.kg^−1^ body weight, Bayer). Then, the heart was exposed through a left intercostal thoracotomy. The left coronary artery was looped by a single nylon suture (7-0) ∼1 mm. The main descending branch was permanently occluded, which resulted in myocardial infarction and, subsequently, CHF. Finally, the heart was quickly repositioned into the chest [28].

### Cardiac Function Assessment

The left ventricular evaluations were performed in anesthetized rats at baseline (4 weeks after surgery) and at the end of study (12 weeks after surgery). Echocardiographic studies were performed with Acuson equipment (Siemens, Mountain View, CA, EUA) with a 13 MHz transducer. Left ventricular dimensions were measured in the M-mode, and left ventricular fractional shortening (FS) was calculated by the formula FS = [(EDD/ESD)/EDD]_x_100, where EDD means left ventricular end-diastolic dimension and ESD left ventricular end-systolic dimension. Ejection fraction (EF) was assessed by Simpson's biplane method at 2-dimensional parasternal long-axis view. All the analyses were conducted according to the American Society of Echocardiography guidelines [29].

### Exercise Capacity

Twenty-four hours after echocardiographic assessment, exercise capacity was measured, which was conducted at baseline (4 weeks after surgery) and at the end of study (12 weeks after surgery). The rats were previously adapted to treadmill exercise before testing. On a treadmill inside of a metabolic chamber coupled to a gas analyzer, rats ran progressively until exhaustion, starting at 6 m.min^−1^ and 3 m.min^−1^ increments every 3 minutes. Peak oxygen uptake (peak VO_2_) was defined as the highest VO_2_ achieved before exhaustion, calculated by the formula peak VO_2_ = [(pO_2_ ambient - pO_2_ at stage)_x_F]/m, where pO_2_ is the partial oxygen pressure measured in the ambient before testing and continuously through each stage inside the chamber, F is the airflow through the chamber (3500 mL.min^−1^), and m is the rat's body mass in kilogram, adapted from Brooks and collaborators [30]. This procedure also provided running distance, in meters.

### Exercise Training

Twenty-hour hours after exercise capacity assessment at baseline, exercise training began. Continuous moderate aerobic exercise training was conducted on a treadmill for 60 minutes at 60% of peak VO_2_, 5 days a week for 8 weeks as described earlier [31]. At the end of the fourth week, training rats underwent another exercise test to adjust the intensity of exercise training. All rats kept sedentary were exposed to treadmill running for 5 minutes, once a week, to maintain their running skills.

### Sacrifice and Collection of Blood and Tissues

Twenty-four hours after peak V0_2_ assessment at the end of the experimental protocol, the rats were quickly sacrificed by decapitation with no prior anesthesia to avoid influences on RAS cascade. Trunk blood was immediately collected into dry (for serum) and cooled tubes (for plasma) with proteases inhibitor cocktail (miniComplete, Roche, USA), separated from total blood as soon as possible, and frozen for posterior analysis. The tissue samples were quickly excised, weighted, and frozen in liquid nitrogen. Thereafter, the samples were stored in a freezer −80° for later analysis.

### ACE and ACE2 Activity Assay

ACE activity was determined in serum, and in skeletal soleus and plantaris muscle by using fluorescent substrates [32]. Frozen skeletal muscle samples were homogenized in 0.1 M Tris-HCl buffer pH 7.0, containing 50 mM NaCl and centrifuged at 1,000×g for 10 minutes. The assays were performed at 37°C in 0.1 M Tris-HCl buffer pH 7.0, containing 50 mM NaCl and 10 µM ZnCl_2_, and captopril 0.5 µL as inhibitor in negative samples. The hydrolysis rate of the intramolecularly quenched fluorogenic substrate Abz-FRK-(Dnp)P-OH (10 uM) incubated with aliquots of homogenate and serum for 30 minutes at 37°C was assessed to obtain ACE enzymatic activity (420 nm λ_em_ and 320 nm λ_ex_, read in 90 cycles). ACE2 activity was determined by the same method described above. However, Abz-APK(Dnp)-OH was used as the fluorescent peptide, in 0.2 M Tris-HCl buffer, 200 mM NaCl, pH 7.5, and DX600 1 mM as the inhibitor. ACE and ACE2 activity are expressed as uF.min^−1.^mg^−1^ of skeletal muscle protein concentration, or uF.min^−1.^mL^−1^ of serum.

### Quantification of Angiotensins

The determination of AngI, AngII, and Ang-(1–7) was quantified by High Performance Liquid Chromatography (HPLC), as previously demonstrated by our group [26]. After centrifugation (10,000×g, 4°C, 20 min), 1 mL of plasma was filtered in Oasis C_18_ columns (Waters, USA), previously activated with methanol (5 mL), tetrahydrofuran (5 mL), hexane (5 mL), methanol (5 mL), and water (10 mL). After activation, the samples were applied into the columns, washed with water and eluted in ethanol/acetic acid/water in the proportion 90%/4%/6%. The eluted fractions were lyophilized and resuspended in 500 µL of mobile phase A (5% acetonitrile in 0.1% orthophosphoric acid) and filtered with 0.22 mm membrane for analysis (HPLC, Shimadzu System, Japan). The angiotensin of each sample was separated on a reversed phase column ODS Aquapor 300 (250×4.6 mm), 7 µ (PerkinElmer's Browlee Columns, USA) using the gradient from 5–35% of mobile phase B (95% acetonitrile in 0.1% phosphoric acid) under a flow of 1.5 mL.min^−1^ for 40 minutes. The angiotensins were identified by comparing them with the retention time of standard angiotensins. Results are expressed as pmol.mL^−1^ of plasma. Skeletal muscle soleus and plantaris samples were weighted and homogenized in 100 mM sodium phosphate buffer pH 7.2, 340 mM sucrose and 300 mM NaCl and protease inhibitor cocktail (Roche, USA). The samples were centrifuged and followed the same sequence as described for plasma angiotensins. Results are expressed as pmol.g^−1^ of tissue.

### Quantification of Protein Expression

The protein expression of ACE and ACE2 in the soleus and plantaris muscles was analyzed using western blot. The frozen samples were homogenized in cell lyses buffer containing 100 mM Tris-HCl, 50 mM NaCl, 1% Triton X-100, and protease inhibitor cocktail (1∶100, Sigma-Aldrich, USA). After centrifugation (10,000 × g, 4°C, 10 min), the pellet was discarded, and the samples were loaded (Laemmli 1∶1, Sigma-Aldrich, USA) and underwent SDS-PAGE in 10% polyacrylamide gels. Equal loading of samples (30 µg) were applied for electrophoresis, and proteins were electro-transferred to nitrocellulose membrane (BioRad Biosciences, USA). The blot membrane was then incubated in a blocking buffer (5% BSA, 10 mM Tris-HCl, pH 7.6, 150 mM NaCl, and 0.1% Tween 20) for 2 hours at room temperature and then incubated overnight at 4°C with mouse anti-ACE (ab11734, 1∶100, Abcam, USA) and rabbit anti-ACE2 (sc-20998 1∶200, Santa Cruz, USA). Binding of the primary antibody was detected with the use of peroxidase-conjugated secondary antibodies, and enhanced chemiluminescence reagents (Amersham Biosciences, USA) were used to visualize the autoradiography. Quantification blot analyses were performed using Image-J software (National Institute of Health, USA), normalized to relative changes in mouse anti-GAPDH (ab9484, 1∶5000, Abcam, USA).

### RNA Isolation and mRNA Quantification

Frozen skeletal muscle samples were homogenized in Trizol, and RNA was isolated according to manufacturer's instructions (Invitrogen Life Technologies, USA). Following extraction, total RNA concentration was quantified using NanoDrop Spectrophotometer (Nano-Drop Technologies, USA) and checked for integrity by EtBr agarose gel electrophoresis. cDNA was synthetized using reverse transcriptase at 70°C for 10 minutes, incubation at 42°C for 60 minutes, and 95°C for 10 minutes. The mRNA expression was assessed by oligonucleotides primers (Exxtend, Brazil) for analysis of the genes AT1a (Sense: 5′-CAC AAC CCT CCC AGA AAG TG-3′; antisense:5′-AGG GCC ATT TTG TTT TTC TG-3′), AT2 (Sense: 5′- GAA CAG AAT TAC CCG TGA CC-3′; antisense: 5′- ATG AAT GCC AAC ACA ACA GC-3′), Mas (Sense: 5′-GAC CAG CCC ACA GTT ACC AGT T-3′; antisense:5′- CCA GGG TTC CCC TTC TGA CT-3′) and reference gene Ciclophilin (Sense: 5′- TGG CAA GCA TGT TGG GTC TTT GGG AAG-3′; antisense: 5′-GGT GAT CTT CTT GCT GCT CTG CCA TTC-3′). Real-time PCRs were run separately, and amplifications were performed by ABI Prism 5700 Sequence Detection System (Applied Biosystems, USA) by using SYBR Green PCR Master Mix (Applied Biosystems, USA). Results were quantified as Ct values, where Ct is defined as the threshold cycle of the polymerase chain reaction at which the amplified product is first detected. Each sample was analyzed in duplicate. Relative quantities of target gene expressions of four groups were compared after normalization to the values of the reference gene (ΔCT). Fold changes in mRNA expression were calculated using the differences in ΔCT values between the two samples (ΔΔCT) and the equation 2-ΔΔCT. Results are expressed in percentages of control (Sham-S).

### Statistical Analysis

Results are presented as mean±SEM. The Student *t* test was conducted to compare infarcted and Sham rats 4 weeks after surgery and, hence, ascertain for CHF. At the end of the protocol (12 weeks after surgery), the groups were compared by 2-way ANOVA (exercise training and CHF as the main factors), with Scheffé test for *post hoc* comparisons. *P* value ≤ 0.05 was accepted as significant.

## Results

### Baseline Measures

Four weeks after surgery, body weight was not significantly different between CHF and Sham rats (386±15 *vs*. 377±11.7 g). Left ventricular end-diastolic diameter (EDD), end-systolic diameter (ESD), fractional shortening (FS), and ejection fraction (EF) were significantly lower in CHF rats compared with that of Sham rats, consistent with cardiac remodeling associated with dilation and dysfunction (Table 1). Functional capacity, expressed by peak VO_2_, and running distance were lower in the CHF rats, indicating exercise intolerance (Table 1). At this point, the rats were divided into sedentary and exercise-trained groups.

**Table 1:** Echocardiographic characteristics and functional capacity of the animals 4 weeks after surgery.

	Sham	CHF	*P*
EDD (mm)	7.36±0.16	9.92±0.22	<0.0001
ESD (mm)	4.01±0.20	9.75±0.45	<0.0001
FS (%)	46.89±1.78	11.97±1.00	<0.0001
EF (%)	80.70±2.46	28.75±2.18	<0.0001
Peak VO_2_ (mL.kg^−1^.min^−1^)	68.90±2.55	59.59±1.90	0.005
Running distance (m)	390.49±23.61	276.49±15.59	<0.0001

CHF, Chronic heart failure; EDD, End-diastolic diameter; ESD, End-systolic diameter; FS, fractional shortening; EF, ejection fraction.

### Effects of Exercise Training on Functional Capacity and Cardiac Function

**Figure 2:**
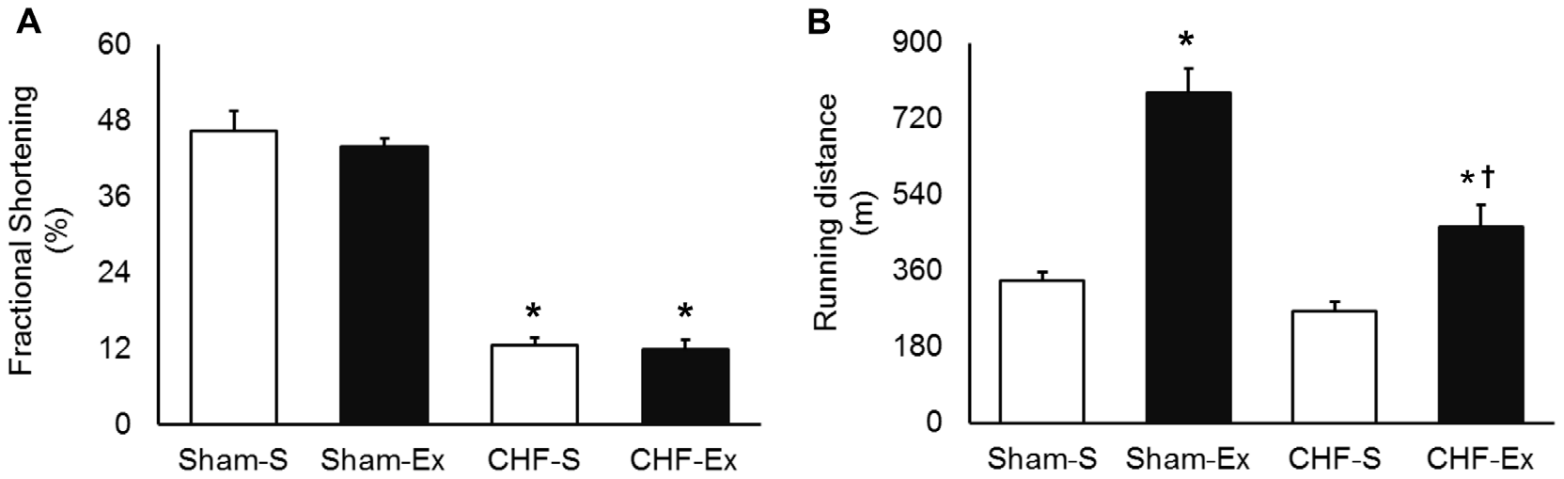
Ventricular function and exercise capacity in sedentary and exercise-trained Sham and CHF rats. CHF, chronic heart failure; -S, Sedentary; -Ex, Exercise-trained. A, Fractional shortening (FS); B, Running distance in Sham and CHF, sedentary and trained rats, 12 week after surgery. *P<0.05 *vs.* Sham-S; †*P*<0.05 *vs.* CHF-S.

Physiological parameters in untrained and exercised-trained Sham and CHF rats are shown in Table 2. There were no significant differences in body weight among groups. The heart and right ventricle mass were significantly increased in CHF rats, and exercise training caused no changes in these parameters (Table 2). Exercise training did not significantly change echocardiographic parameters or peak VO_2_ in CHF rats. However, exercise training significantly increased running distance in both Sham-operated and CHF rats but caused no change in FS (Fig. 2).

**Table 2:** Physiological parameters in sedentary and exercised-trained Sham-operated and CHF rats.

	Sham-S	Sham-Ex	CHF-S	CHF-Ex
Body weight (g)	452.13±11.96	424.97±7.93	451.83±17.21	423.59±13.47
Heart (g)	1.12±0.04	1.10±0.03	1.46±0.07*	1.52±0.06*
RV (g)	0.23±0.01	0.23±0.01	0.42±0.05*	0.43±0.02*
EDD (mm)	7.24±0.24	7.70±0.21	11.55±0.32*	11.30±0.32*
ESD (mm)	3.72±0.25	4.54±0.19	10.13±0.36*	9.43±0.60*
EF (%)	81.83±2.73	79.92±1.42	29.73±2.69*	28.37±3.18*
Peak VO_2_ (mL.kg^−1^.min^−1^)	65.86±2.24	73.96±2.39*	55.99±2.62	62.21±2.58

CHF, chronic heart failure; -S, Sedentary; -Ex, Exercise-trained; RV, right ventricle mass; EDD, End-diastolic diameter; ESD, End-systolic diameter; EF, left ventricular ejection fraction.

*P<0.05 *vs.* Sham-S.

### Effects of Exercise Training on Circulating RAS

**Figure 3:**
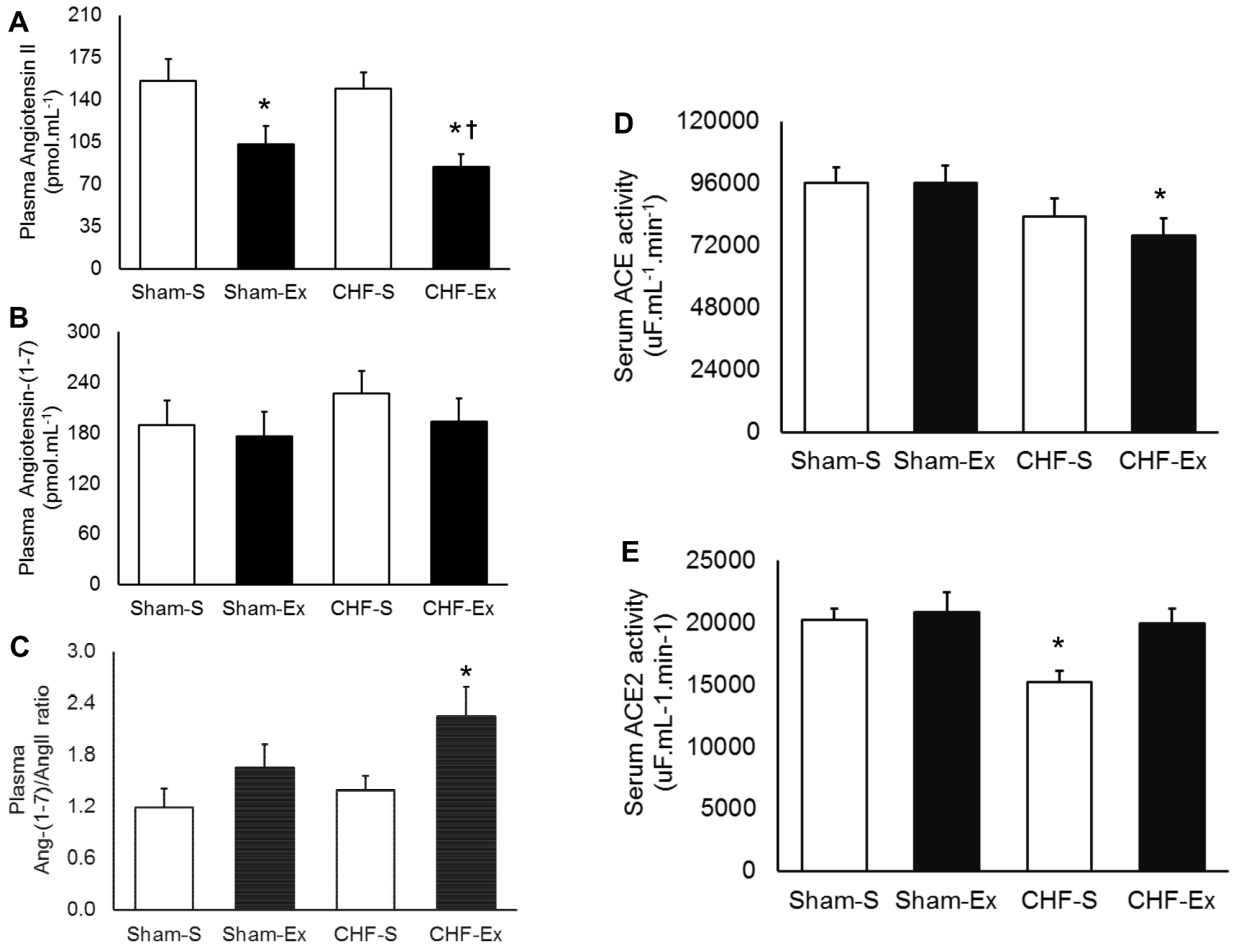
Circulating renin angiotensin system (RAS) in sedentary and exercise-trained Sham and CHF rats. CHF, chronic heart failure; -S, Sedentary; -Ex, Exercise-trained. A, Angiotensin II; B, Angiotensin-(1–7); C, Ang-(1–7)/AngII ratio; D, ACE activity; E, ACE2 activity. *P<0.05 *vs.* Sham-S; †*P*<0.05 *vs.* CHF-S.

CHF did not change serum ACE activity, but caused a 25% reduction in ACE2 activity (Fig. 3, *P* = 0.04). Exercise training significantly decreased ACE activity in the CHF rats (*P* = 0.05), and restored the ACE2 activity towards the levels found in Sham-operated rats (Fig. 3). No significant changes in ACE and ACE2 activity occurred in the Sham-Ex. In regard to plasma angiotensins, we found no significant changes in plasma AngII levels in CHF rats. Exercise training provoked a significant reduction in AngII levels in both Sham (33%, *P* = 0.03) and CHF rats (43%, *P* = 0.007) (Fig. 3). Plasma AngI and Ang-(1–7) were unchanged by CHF or exercise training (Table 3 and Fig. 3). However, exercise training significantly increased the Ang-(1–7)/AngII ratio in CHF rats (Fig. 3). No changes were found in the Sham-Ex.

**Table 3:** Plasma and skeletal muscle AngI in sedentary and exercise-trained Sham-operated and CHF rats.

	Sham-S	Sham-Ex	CHF-S	CHF-Ex
Plasma AngI (pmol.mL^−1^)	88.79±8.22	75.00±9.73	97.76±12.17	70.40±12.25
Soleus AngI (pmol.g^−1^)	39.18±9.68	28.50±2.96	59.79±16.98	46.46±8.44
PlantarisAngI (pmol.g^−1^)	49.19±9.01	38.88±11.76	67.70±14.90*	39.94±5.74†

CHF, chronic heart failure; -S, Sedentary; -Ex, Exercise-trained; AngI, Angiotensin I.

*P<0.05 *vs.* Sham-S;

†*P*<0.05 *vs.* CHF-S.

### Effects of Exercise Training on RAS in the Soleus Muscle

**Figure 4:**
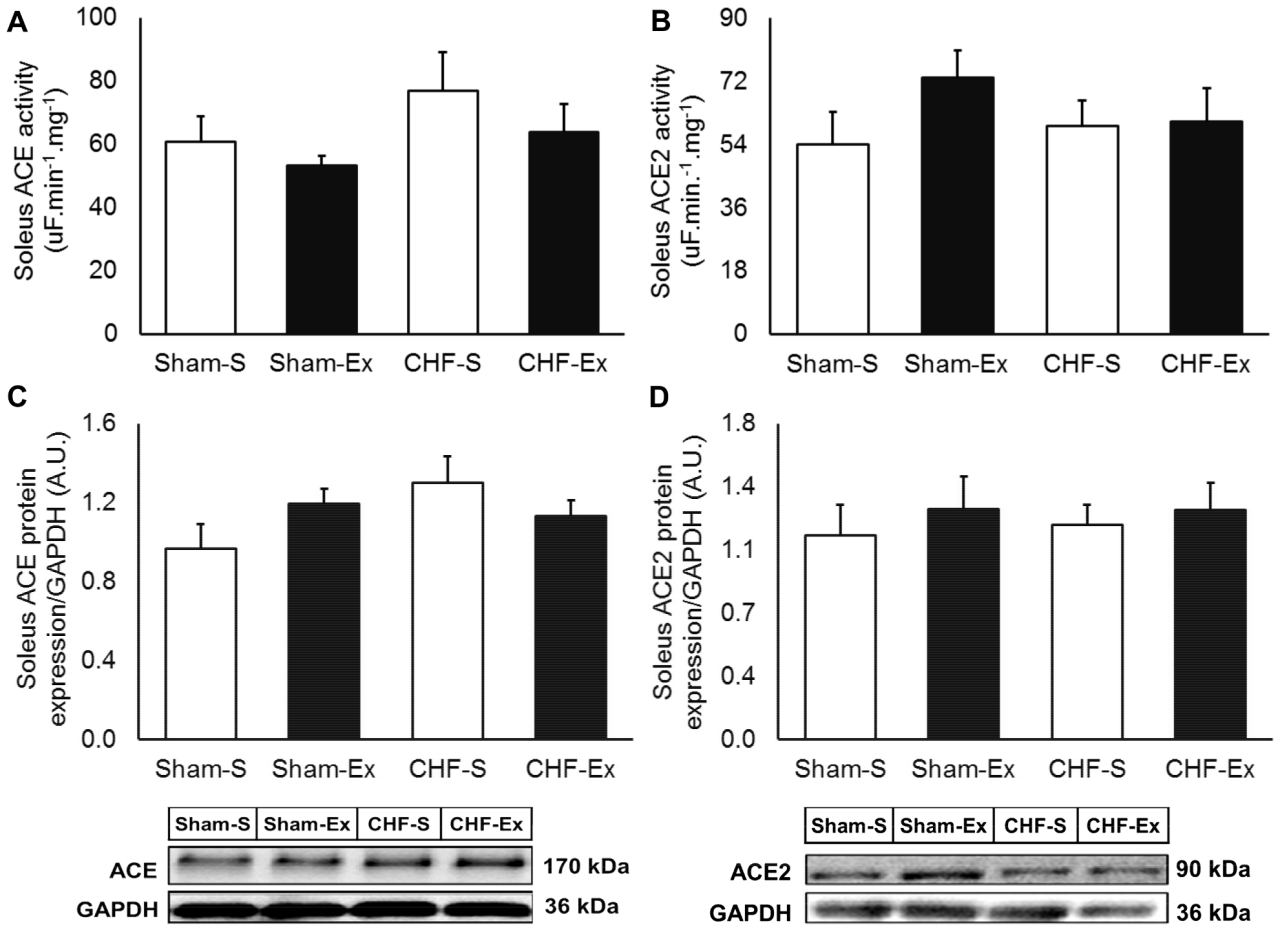
Soleus muscle ACE and ACE2 in sedentary and exercise-trained Sham and CHF rats. CHF, chronic heart failure; -S, Sedentary; -Ex, Exercise-trained; ACE, Angiotensin Converting Enzyme. A, ACE activity; B, ACE2 activity; C, ACE protein expression; D, ACE2 protein expression.

**Figure 5:**
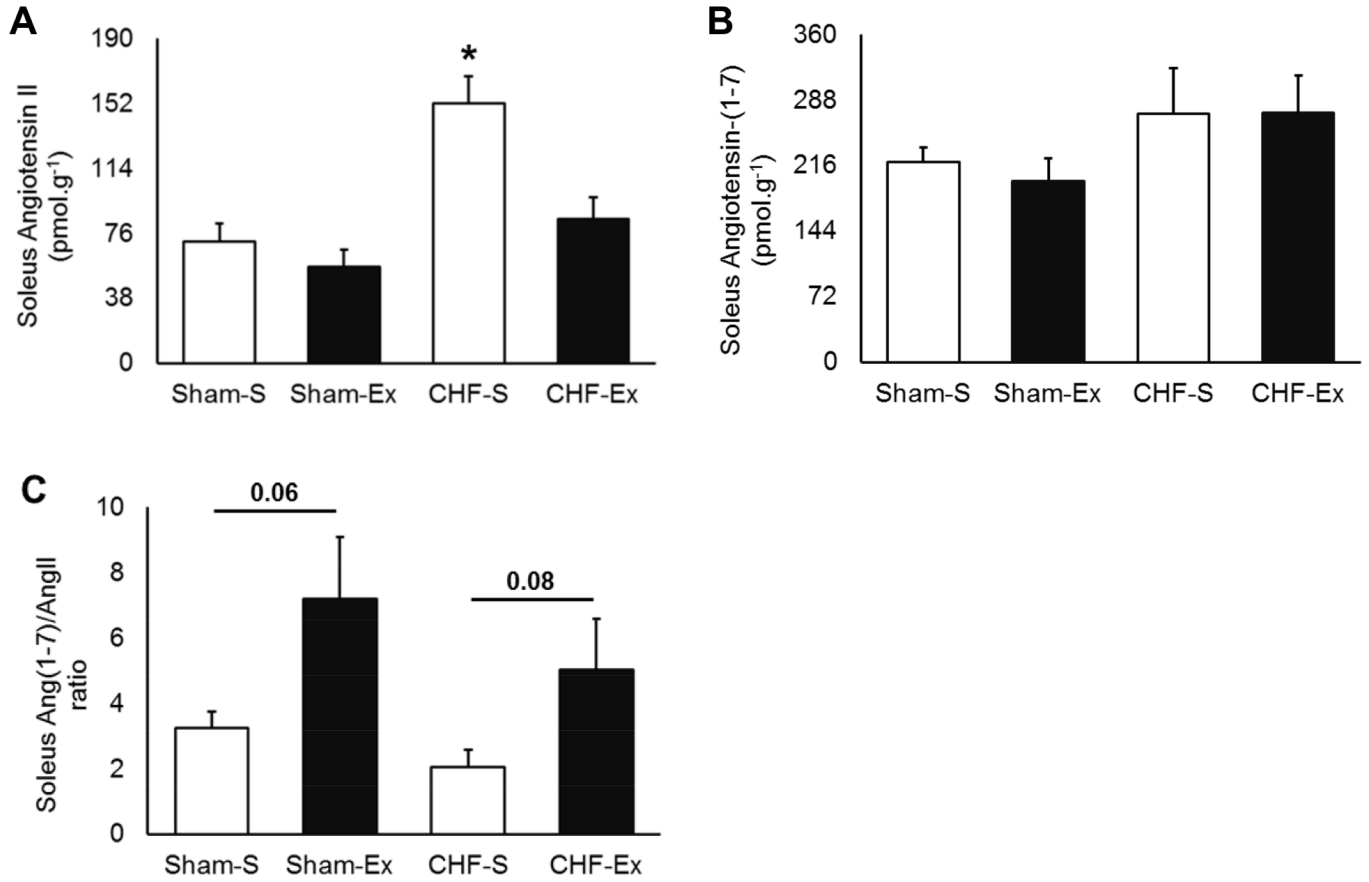
Soleus muscle angiotensins in sedentary and exercise-trained Sham and CHF rats. CHF, chronic heart failure; -S, Sedentary; -Ex, Exercise-trained. A, Angiotensin II; B, Angiotensin-(1–7); C, Ang-(1–7)/AngII ratio. *P<0.05 *vs.* Sham-S.

**Figure 6:**
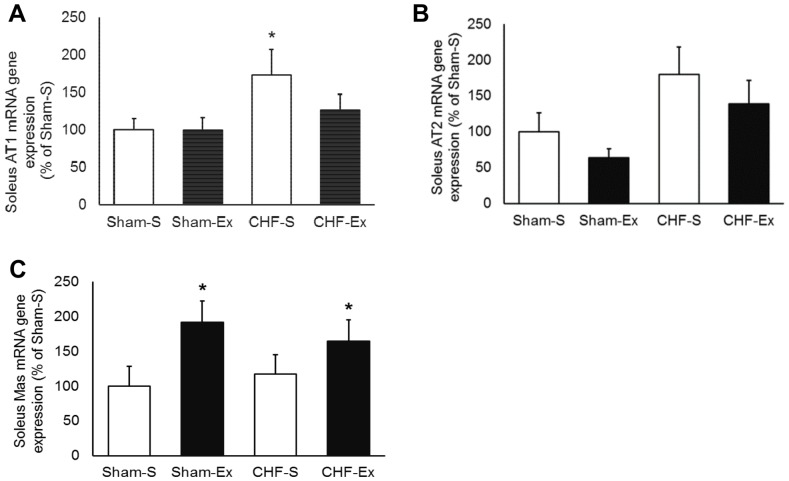
Soleus muscle angiotensin receptors in sedentary and exercise-trained Sham and CHF rats. CHF, chronic heart failure; -S, Sedentary; -Ex, Exercise-trained. A, AT1 mRNA gene expression; B, AT2 mRNA gene expression; C, Mas mRNA gene expression. *P<0.05 *vs.* Sham-S.

CHF or exercise training did not change ACE activity and protein expression in soleus muscle (Fig. 4). CHF provoked a 2.1-fold increase in AngII concentration in the soleus muscle (*P* = 0.001), and exercise training decreased it to the normal levels (Fig. 5). There were no significant differences in AngI among groups (Table 3). The AT1 receptor gene expression in the soleus muscle was significantly increased in CHF rats (*P* = 0.04, Fig. 6). Exercise training normalized these levels. AT2 gene expression was not different among groups. Similarly, no changes in Ang-(1–7) concentration were found among groups (Fig. 5). Exercise training tended to increase Ang-(1–7)/AngII ratio in Sham-operated (*P* = 0.06) and CHF (*P* = 0.08) rats (Fig. 5). Exercise training increased Mas gene expression in both Sham and CHF rats (*P* = 0.02) (Fig. 6).

### Effects of Exercise Training on RAS in Plantaris Muscle

**Figure 7:**
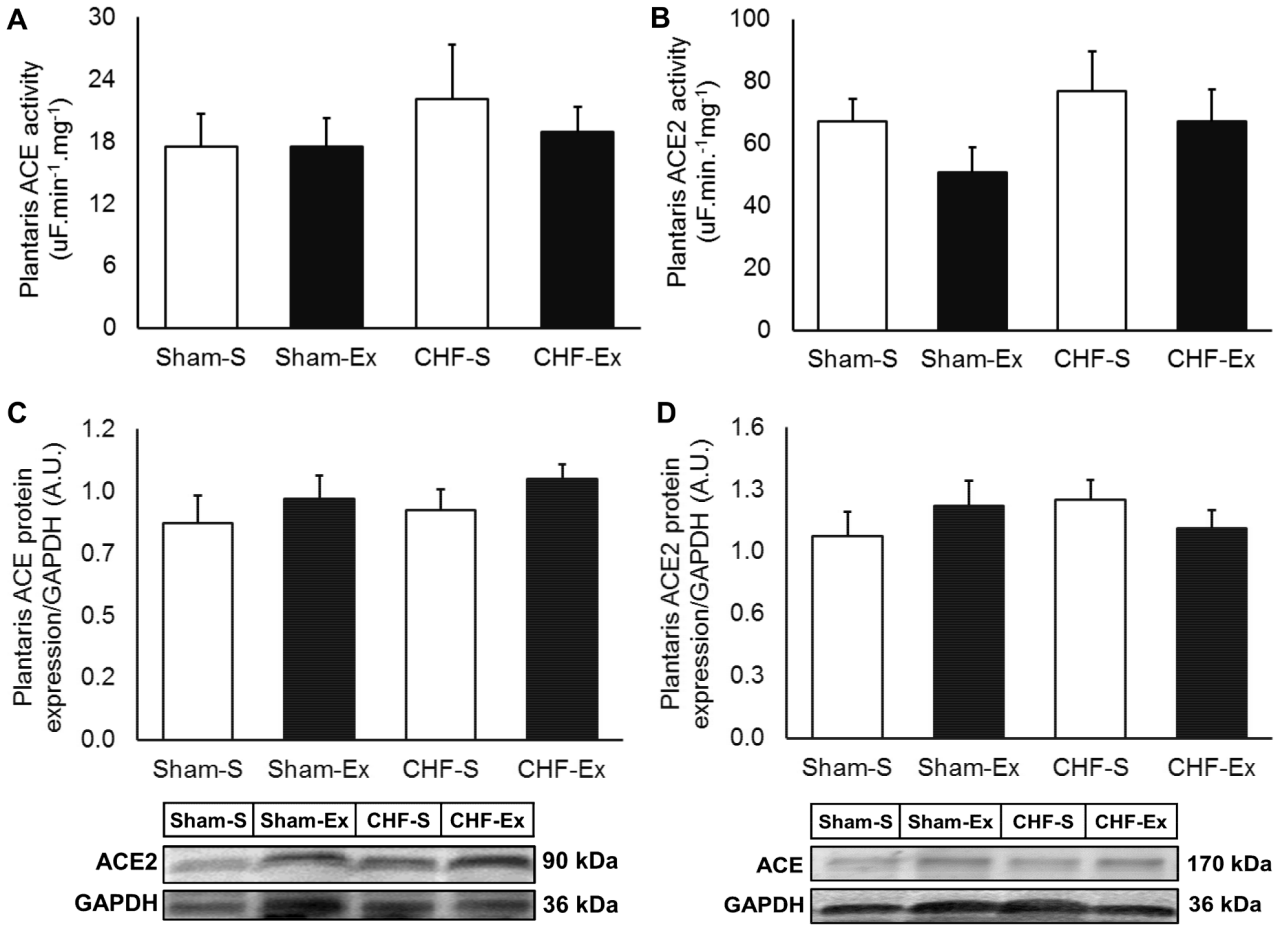
Plantaris muscle ACE and ACE2 in sedentary and exercise-trained Sham and CHF rats. CHF, chronic heart failure; -S, Sedentary; -Ex, Exercise-trained; ACE, Angiotensin Converting Enzyme. A, ACE activity; B, ACE2 activity; C, ACE protein expression; D, ACE2 protein expression.

**Figure 8:**
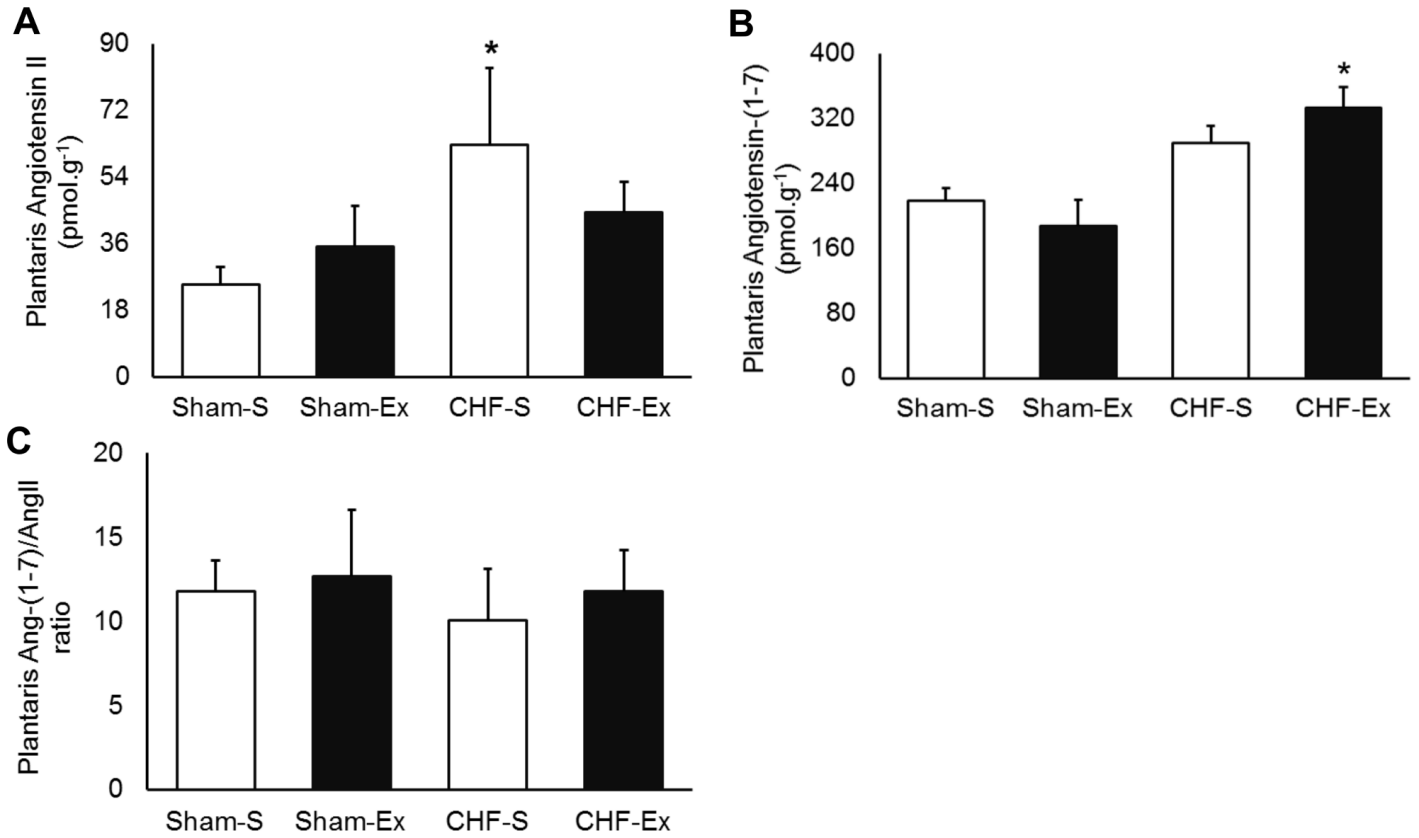
Plantaris muscle angiotensins in sedentary and exercise-trained Sham and CHF rats. CHF, chronic heart failure; -S, Sedentary; -Ex, Exercise-trained. A, Angiotensin II; B, Angiotensin-(1–7); C, Ang-(1–7)/AngII ratio. *P<0.05 *vs.* Sham-S.

**Figure 9:**
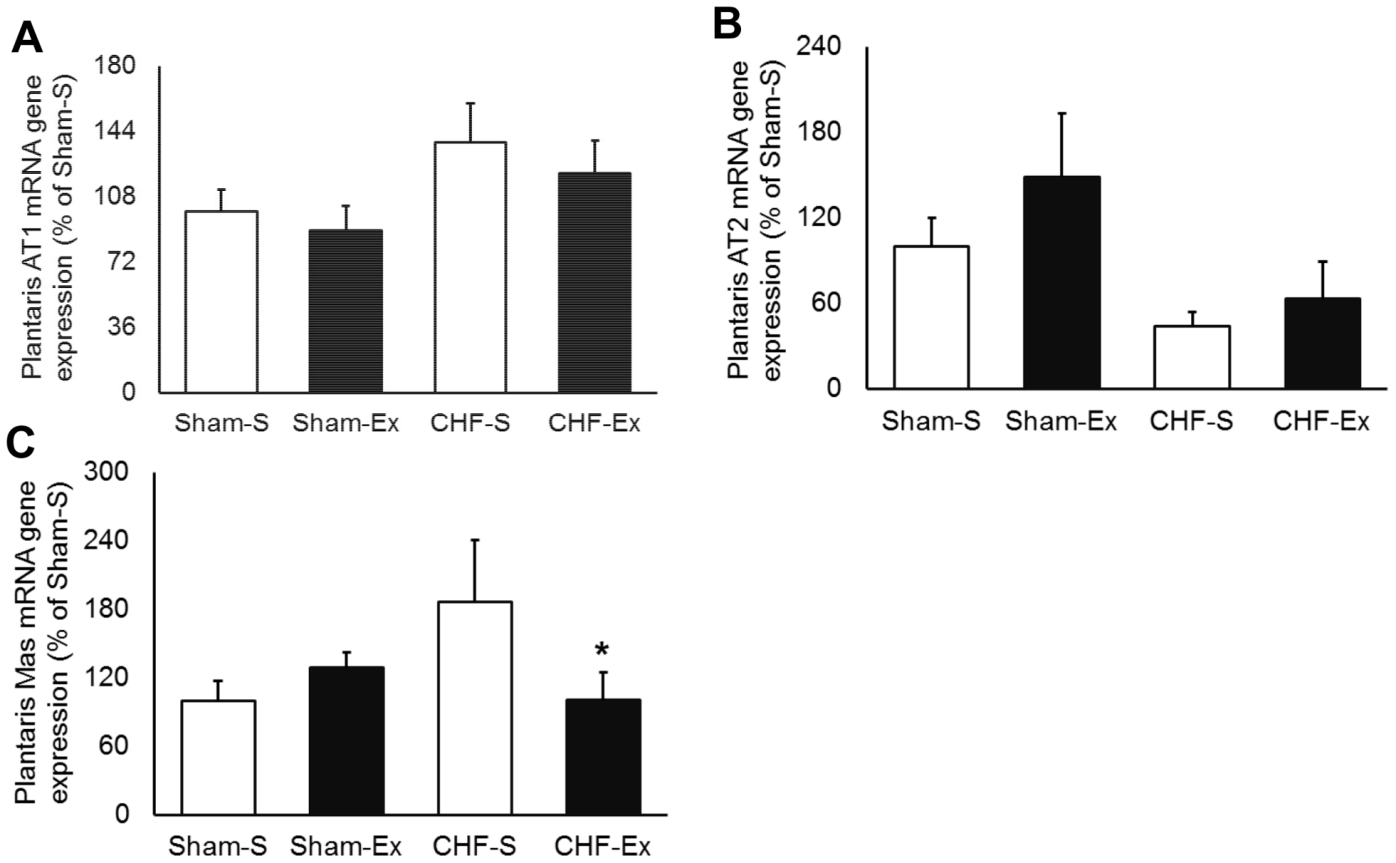
Plantaris muscle angiotensin receptors in sedentary and exercise-trained Sham and CHF rats. CHF, chronic heart failure; -S, Sedentary; -Ex, Exercise-trained. A, AT1 mRNA gene expression; B, AT2 mRNA gene expression; C, Mas mRNA gene expression. *P<0.05 *vs.* Sham-S.

In regard to plantaris muscle, CHF and exercise training unchanged ACE activity and protein expression (Fig. 7). In contrast, AngI and AngII in the plantaris muscle were 1.5- and 2.5-fold increased of CHF rats (Table 3, Fig. 8). CHF and exercise training did not change AT1 or AT2 receptors (Fig. 9). Exercise training decreased AngI and AngII towards normal levels (Table 3, Fig. 8). Exercise training significantly increased Ang-(1–7), but caused no significant changes in Ang-(1–7)/AngII ratio (Fig. 8). There were no differences in ACE2 activity or protein expression among groups (Fig. 7). Exercise training significantly reduced the Mas gene expression in CHF (Fig. 9).

## Discussion

The main and new findings of the present study are that exercise training in an ischemic model of CHF: 1) Normalizes AngII concentration in soleus and plantaris muscle; 2) Decreases AT1 receptors towards normal levels in soleus muscle; 3) Increases Ang-(1–7)/AngII ratio in plasma and soleus; and 4) Increases Mas receptor mRNA expression in soleus muscle.

Our study confirms previous studies [10], [25] that demonstrated that exercise training causes remarkable changes in circulating RAS in CHF. The reduction in circulating ACE activity and AngII concentration in CHF rats has important implications. Firstly, the improvement in arterial baroreflex control of renal sympathetic nerve activity in CHF rats depends on the reduction in plasma AngII [31]. Mousa and collaborators [10] elegantly demonstrated that administration of AngII to maintain its levels near those found in untrained CHF restrained the amelioration in arterial baroreflex sensitivity in exercise-trained CHF rabbits. Secondly, AngII increases sympathetic nerve activity [10], [33]. This knowledge may predict that exercise training decreased sympathetic outflow in our study. In fact, sympathetic deactivation has been consistently reported after exercise training in CHF [10], [23], [31]. Thirdly, both the improvement in baroreflex sensitivity and the reduction in sympathetic activity are associated with better prognosis in CHF [34], [35].

In CHF patients, there is an association between serum ACE2 and the severity of this syndrome [36]. A possible explanation for this response is that AngII provokes ACE2 shedding mediated by TACE/ADAM-17, which increases serum ACE2 activity [37]. In conformity to a previous study on the same experimental model [38], we found that serum ACE2 activity was reduced in CHF. The contrast between serum ACE2 in humans with CHF and the experimental model of CHF is not clear. However, it could be speculated that the pharmacological inhibition of ACE usually prescribed for humans with CHF causes a compensatory increase in serum ACE2 activity. In fact, some investigators have previously observed an association between serum ACE inhibition and ACE2 increase [39]. Exercise training significantly decreased serum ACE activity and increased serum ACE2 activity. This finding reinforces the inverse association between ACE and ACE2 activity.

Despite the fact that exercise training increases serum ACE2 activity towards normal levels, no significant changes in circulating Ang-(1–7) concentration were found. Since AngII is the major substrate for the production of Ang-(1–7), it is possible to anticipate that the reduction in AngII concentration limited the formation of Ang-(1–7). All together, these findings indicate that exercise training causes a switch in circulating ACE-AngII towards an increase in ACE2-Ang-(1–7) axis in CHF rats, which may render the cardiovascular system less susceptible to the deleterious actions of AngII [40].

We hypothesized that exercise training would cause a shift in RAS in skeletal muscle towards the ACE2-Ang-(1–7)-Mas axis. Our findings confirm in great part this hypothesis. Exercise training decreased AngII in both soleus and plantaris muscle in the ischemic model of CHF. In addition, exercise training tended to increase the Ang-(1–7)/AngII ratio, which suggests an imbalance in the RAS towards the ACE2-Ang-(1–7)-Mas receptor axis. The reduction in the AngII concentration and AT1 receptor gene expression in skeletal muscle is an important issue. Previous studies show that AngII is involved in inflammation and oxidative stress [13], [41]. These alterations contribute to muscle catabolism and apoptosis in CHF [12]–[14], [42], [43]. Our study provides no information on muscle protein synthesis and degradation. Future investigations should focus on the contribution of ACE-AngII-AT1 receptor axis in the amelioration in skeletal myopathy.

The mechanisms by which exercise training decreased AngII concentration in skeletal muscle in CHF rats is out of the scope of the present study. However, it is possible that exercise training reduced AngII uptake in the soleus muscle [44], because AT1 expression was substantially lower in the exercise-trained rats. In plantaris muscle, exercise training also reduced AngII concentration, but caused no changes in AT1 expression. These findings raise the question that the mechanisms underlying the reduction in AngII in plantaris muscle and soleus muscle are different. Someone could suggest that the reduction in AngII in the plantar muscle is due to the decrease in AngI cleavage. In fact, we found that AngI concentration in plantar muscle was significantly lower in exercise-trained CHF compared to that in untrained CHF rats.

Our data clearly demonstrate that there is a mismatch between circulating and skeletal muscle RAS. Elevation in AngII concentration was found in the skeletal muscle, but not in the circulation. These findings are consistent with those of previous studies that show that local RAS becomes activated in tissues in CHF [5]–[8]. The increase in the plasma AngII concentration seems to occur only in the late stage of CHF [3], [4]. ACE was not altered in the skeletal muscle, which suggests alternative local pathways of AngII production involving other enzymes as Prolyl-endopeptidase, Neprilysin and Chymase [45]–[47], especially in the plantaris muscle. Exercise training reduced AngII concentration in both plasma and skeletal muscle. Exercise training reduces ACE and restores ACE2 activity in the circulation, without changing ACE and ACE2 activity or protein expression in skeletal muscle. Other investigators reported that electric stimulated C2C12, a strategy to mimic *in vitro* exercise effects, caused no changes in skeletal muscle ACE gene expression [48].

Previous studies demonstrated that the AT1 receptor was increased in the vasculature of patients with ischemic heart disease [49], and in local tissues in experimental models of CHF [5], [7], [10]. In our study, we found increased AT1 receptor mRNA in soleus muscle in CHF rats. This increase in AT1 receptors may be associated with elevation in sympathetic nerve activity. In a previous study, isoproterenol administration increased AT1 receptor expression [50]. Exercise training reverted AT1 receptor gene expression towards normal levels, which may be linked to reduction in sympathetic activation. We and others have consistently found a reduction in sympathetic nerve activity in heart failure [10], [23], [31].

We found no changes in AT2 receptors in our study. The AT2 gene expression in both soleus and plantar muscles was not altered in CHF rats. Similar findings have been reported in patients with heart failure. Some investigators found no AT2 gene expression in skeletal muscle in patients with CHF [51]. In our study, exercise training caused no changes in AT2 receptors in either normal controls or CHF rats.

Our study demonstrates for the first time that exercise training changes the Ang-(1–7) and Ang-(1–7)/AngII ratio and Mas receptor expression in skeletal muscle in CHF rats. These are remarkable effects provoked by exercise training. Ang-(1–7) is a potent vasodilator as are other interventions in the treatment of heart failure, and in the muscle level, Ang-(1–7)/Mas improves insulin sensitivity by increasing Akt phosphorilation [19].

### Limitations

We recognize limitations in our study. We only studied mRNA expression of angiotensin receptors. Thus, someone could argue that there is no guarantee that the changes in gene expression have been translated into protein expression. Our study provides no direct information regarding the implications of RAS modifications in the function of skeletal muscle. The increase in running distance is suggestive of improvement in functional capacity, but not sufficient to demonstrate amelioration in skeletal muscle function.

### Perspectives

The antagonism of the RAS and sympathetic outflow is the target in the treatment of heart failure. The present study shows that exercise training provokes profound changes in circulating and skeletal muscle RAS. The switch from AngII/AT1 to Ang-(1–7)/Mas axis in the skeletal muscle may have implications in the skeletal myopathy in heart failure. Growing evidence shows that AngII is involved in the skeletal muscle pathophysiology of heart failure. Finally, these findings strengthen the recommendation of exercise training in the treatment of CHF.

In conclusion, exercise training improves RAS in the Ang-(1–7)-Mas axis in skeletal muscle. This effect of exercise training seems to depend on skeletal muscle metabolic characteristics. The changes in circulating RAS do not necessarily reflect the changes occurring in skeletal muscle RAS.
